# Information Dynamics of Whole Genome Adaptation

**DOI:** 10.4236/ns.2014.615110

**Published:** 2014-10

**Authors:** William Hercules, James Lindesay, Tshela E. Mason, Georgia M. Dunston

**Affiliations:** 1 Computational Physics Laboratory, Howard University, Washington DC, USA; 2 National Human Genome Center, Howard University, Washington DC, USA; 3 Department of Microbiology, Howard University, Washington DC, USA

**Keywords:** Genomic Information, Population Adaptation, Genomic Homeostasis, Genome-Environment Interactions

## Abstract

The human genome is a complex, dynamic information system that encodes principles of life and living systems. These principles are incorporated in the structure of human genome sequence variation and are foundational for the continuity of life and human survival. Using first principles of thermodynamics and statistical physics, we have developed analogous “genodynamic tools” for population genomic studies. Characterizing genomic information through the lens of physics has allowed us to develop energy measures for modeling genome-environment interactions. In developing biophysical parameters for genome-environment homeostasis, we found that stable genomic free energy trades off low genomic energy (genomic conservation and increased order) and high genomic entropy (genomic variation) with an environmental potential that drives the variation. In our approach, we assert that common variants are dynamic sites in the genome of a population and that the stability of whole genome adaptation is reflected in the frequencies of maintained diversity in common variants for the population in its environment. In this paper, we address the relativity of whole genome adaptation towards homeostasis. By this we mean that adaptive forces are directly reflected in the frequency distribution of alleles and/or haplotypes of the population relative to its environment, with adaptive forces driving the genome towards homeostasis. The use of genomic energy units as a biophysical metric in DNA sequence variation analyses provides new insights into the foundations of population biology and diversity. Using our biophysical tools, population differences directly reflect the adaptive influences of the environment on populations.

## 1. Introduction

The completion of the Human Genome Project introduced a new knowledge system for decoding biology and population diversity based on information encoded in the structure of DNA sequence variation. The Biophysics Research and Development Group in the National Human Genome Center at Howard University has applied first principles of thermodynamics and statistical physics in studying common variants (*i.e.*, polymorphisms) as dynamic sites in the genome. This has allowed us to model the genome as a dynamic information system with potentialities for the expression of life in and through all human biology-past, present, and future. These potentialities provide flexibility needed for human survival in varying environments. We submit that on a population level, human survival manifests as maintained diversity associated with whole genome adaptation to the environment. Thus, this paper focuses on genomic information dynamics of whole genome adaptation expressed globally in population diversity. This focus is predicated on the supposition of the human genome as a structured dynamic information system that governs the development, function, and expression of life at all levels of the biological hierarchy, from whole genomes in cells to whole populations.

Our objective in this short population genomics report is to introduce the information dynamics of whole genome adaptation towards homeostasis in population biology. Population *genomics*, the large-scale comparison of DNA sequences of populations focuses on the whole genome as the functional unit. This is in contrast to population *genetics* which focuses on individual genes. In our population genomic studies, the population is defined by the pattern of maintained diversity in the spectrum of allele and/or haplotype frequencies for the population in its environment. This is unlike many conventional studies in population genetics where the population is defined by some subset of the spectrum of allele(s) and/or haplotype(s) most frequent in the population. The constraints of the latter reveal our definition of the population to be more inclusive and comprehensive.

In modeling whole genome adaptation towards homeostasis of the population in its environment, we consider genome-environment interaction as inextricable. Moreover, changes in the distribution of allele and haplotype responses to the environment directly reflect adaptive forces on the population. Thus, adaptive forces drive the genome towards homeostasis. We further note that modeling the information dynamics of whole genome adaptation to environmental stressors does not require detailed knowledge of the molecular mechanisms underlying biology.

## 2. Genomic Information

As a structured, dynamic information system, the information dynamics of the whole genome determines adaptation in a given environment. Genomic information is encoded in patterns of sequence variation. The information content of a genomic distribution is the maintained order reflected in the patterns of sequence variation. This maintained order can be related to the disorder that is parameterized by the entropy *S* of the distribution given by

(1) $$S=-\sum_{j}p_{j}\log_{2}\;p_{j},$$

where *p_j_* represents the probability of the occurrence of a variant of type *j* within the distribution. The degree of order, quantified by the information content is defined using the equation

(2) $$\mathrm{IC}=S_{\max}-S$$

where *IC* is the information content of the distribution, *S* is the previously defined entropy of the distribution and *S*_max_ represents the maximum entropy that results when all possible variations occur with equal likelihood. Using this formula a distribution with zero information content has maximum disorder, *S* = *S*_max_ whereas a distribution with a normalized information content equal to one contains maximum order, *S* = 0. As an information system the human genome must be dynamic, this means that it varies in expression over time and environment. We can therefore model the genome as an information system defined by patterns of sequence variation whose dynamics correlate using genomic energy units. The adaptive forces are determined by how these patterns change as a function of the environmental parameters. The adaptive forces are what drive the changes in the patterns of sequence variation. These forces are directly related to the dynamics of the genomic energies, this therefore ties the dynamics of genomic information to adaptation.

## 3. Population Adaptation

Living systems are inherently adaptive. For our purposes, adaptation is the process by which a population approaches homeostasis in a given environment. Here we will not consider mutations or evolutionary changes in the genome, but rather genomic adaptation involving changes in the frequency distribution of alleles or combinations of alleles (*i.e.* haplotypes). Since the environments in which life exist cover a range of physical, chemical, biological, psychological, and sociological conditions, adaptation is imperative for the continuation of life and human survival. From their original habitat, human populations migrated out of Africa to inhabit all geographic areas spanning the globe.

The movement of human population groups between habitats exposes the migrating groups to different ecological niches which include geography, climate, vegetation, food sources and availability, microorganisms, and predators. That human populations have survived in different ecological environments is evidence of whole genome adaption as a principle of life and living systems. It is therefore assumed that relatively small adaptive changes occur in populations for them to remain viable in the process of whole genome adaptation towards homeostasis.

Past migrations provide a natural laboratory within which whole genome-environment interactions can be examined, particularly if suitable parameters characterizing the environment can be found. Our research interests in analyzing genome-environment interactions were tremendously augmented by the availability of comprehensive haplotype maps (HapMap) of sequence variation in different continental populations, produced as powerful reference resources to the Human Genome Project’s complete sequencing of a reference human genome. The availability of HapMap data on population-associated patterns in sequence variation in different global populations provided the resources to investigate adaptive forces that have influenced patterns of sequence variation in natural populations. Our group has developed biophysical metrics that correlate information dynamics with genomic energy units. We submit that adaptive forces determine how these patterns change as a function of the environmental parameters and are what drive changes in the frequency patterns of sequence variation. Moreover, directly relating these adaptive forces to the dynamics of the genomic energies ties the genomic information dynamics to population adaptation. Ultimately, a population reaches homeostasis characterized by a specific distribution of single nucleotide polymorphisms (SNPs) and SNP haplotypes associated with its adaptation to that environment. Even for a population in homeostasis, continual environmental challenges require maintained diversity.

Population homeostasis involves the maintenance of a state of optimal interaction of its genome with the environment. In contrast with a physical system in thermal equilibrium with its environment, genomic homeostasis maintains a state of *dis-equilibrium*. However, a state of genomic homeostasis can be expressed in terms of state variables parameterizing the information dynamics for the genome in that environment. We define genomic free energy by

(3) $$F_{\text{genome}}=U_{\text{genome}}-T_{E}S_{\text{genome}}$$

where *U*_genome_ represents a genomic energy whose minimization drives the genomic distribution towards information conservation; *T_E_* is the environmental potential driving genomic diversity, and *S*_genome_ is the genomic entropy which quantifies the genomic variation. This indicates that a population with given genomic free energy *F*_genome_ has established a balance between the allelic conservation and allelic variation of the population. The parameterization of genomic informatics in terms of genomic free energy makes a statement inherent for living systems that diversity and conservation are inextricable. A population in homeostasis maintains minimal genomic free energy, expressed as

(4) $${\mathrm{dF}}_{\text{genome}}=0.$$

Minimal genomic free energy implies that the genomic informatics remains unchanged under small variations in populations (*i.e.* Hardy-Weinberg equilibrium) or small changes in environmental parameters. Once genomic energy units that parameterize the information dynamics of the population have been established in a given environment, these measures can then be used to determine adaptive forces acting upon *individual* alleles and haplotypes. In addition, these measures can be used to determine adaptive forces upon population averaged distribution of alleles. In general, collective adaptive forces drive the genomic distribution towards homeostasis. Using these tools, the information dynamics of whole genome adaptation can be modeled. Modeling the information dynamics of whole genome adaptation to the environment abrogates the need for detailed prior knowledge of the underlying biological mechanisms associated with adaptation.

## 4. Pathogens as Stressors for Modeling Genomic Adaptation

Pathogens are one type of environmental stressors that impact genomic adaptation. Information dynamic tools can be used to model whole genome response to environmental stressors.

An environmental stressor of global significance and particular interest is the plasmodium parasite, the causative agent of malaria. Malaria is considered one of the strongest known adaptive forces for selection in recent history due to the number of selective signatures present in the human genome [1]. Differences between population groups with regard to malaria susceptibility have been of great interest within recent years, and these differences in susceptibility have been linked to differences at the genetic level [2]. Differences have also been found in sympatric populations, where the populations share the same environment but suffer different susceptibility rates and burden of disease [3] [4]. Some of the genetic variation that has arisen in response to malaria infection has been in genes that either directly or indirectly modulates the host immune response, or that are responsible for the regulation of host-parasite interactions [5]. This co-adaptation in both the human and the parasite illustrates the influence the parasite has on the host genome. This has resulted in significant shaping of the host genome. Understanding the information dynamics of whole genome adaptation encoded in human and parasite genomes can offer great insight into the interplay between host-parasite interactions. The genomic information of the parasite may provide insight into the methods utilized by the parasite to produce infection in the host, as well as evasion of the host immune response. Additionally, we expect that the human genome has impacted the parasite’s genome and its ability to survive. This clearly illustrates an instance of co-adaptation. Thus host-parasite relationships offer intriguing opportunities to explore mutual information dynamics in co-adaptation.

## 5. Conclusion

We have used the parameterization of genomic information to derive a genomic energy metric related to genomic free energy. Once genomic energy units that parameterize the dynamics of the population have been established in a given environment, these measures can then be used to determine adaptive forces acting upon individual alleles or haplotypes. In general, collective adaptive forces drive the genome towards homeostasis. Using these tools the information dynamics of whole genome adaptation can be modeled.
